# The Myth of Zero-Sum Responsibility: Towards Scaffolded Responsibility for Health

**DOI:** 10.1163/17455243-20233725

**Published:** 2023-09-07

**Authors:** Neil Levy, Julian Savulescu

**Affiliations:** Uehiro Centre for Practical Ethics, University of Oxford, Oxford, United Kingdom; Centre for Biomedical Ethics, National University of Singapore, Singapore

**Keywords:** blame, group agents, public health, responsibility

## Abstract

Some people argue that the distribution of medical resources should be sensitive to agents’ responsibility for their ill-health. In contrast, others point to the social determinants of health to argue that the collective agents that control the conditions in which agents act should bear responsibility. To a large degree, this is a debate in which those who hold individuals responsible currently have the upper hand: warranted appeals to individual responsibility effectively block allocation of any significant degree of responsibility to collective agents. We suggest that a different understanding of individual responsibility might lead to a fairer allocation of blame. Scaffolded agency is individual agency exercised in a context in which opportunities and affordances are structured by others. Appeals to scaffolded agency at once recognize the role of the individual and of the collective agents who have put the scaffolds in place.

Lifestyle is a very significant contributor to the morbidity and mortality associated with all the big killers in developed countries (cardiopulmonary disease; cancer; stroke). For example, it has been estimated that 40% of cancers diagnosed in the UK could be prevented by changes in lifestyle (Davis 2018). Put differently, patients are often (in some sense) responsible for the development of their cancers: had they behaved differently (smoked less; exercised more; eaten better) they would have been much less likely to develop the disease (Yoon et al. 2014). But healthcare resources are in limited supply, and we must make decisions regarding how they are to be distributed.

We may use a variety of different criteria to decide who gets the bed, the ventilator, or the organ. Most of these criteria are purely medical: for example, we may decide between patients on the basis of who might be expected to benefit more from the resource, with ‘benefit’ understood in medical terms (perhaps measured in QALYS). Sometimes, however, we cannot decide between patients on the basis of such criteria. In cases like this, a number of ethicists advocate the use of responsibility as a criterion (Segall 2010; McMillan 2019; Cappelen & Norheim 2005).

Responsibility can – in principle – play the role of grounding ethically justifiable allocation conditions only if it is linked to what agents *deserve*. Were an agent blamelessly to cause their own need for healthcare resources (for example, causing themselves to develop a cancer of the mouth by gargling with an alcohol-based mouthwash recommended to them by their dentist), there would be no grounds for giving them lower priority when it comes to the allocation of scarce resources. Advocates of using responsibility as a criterion for the allocation of resources maintain that those who are responsible are (somewhat) less deserving of resources than those who are not, and only moral responsibility can underwrite this appeal to desert.

Moral responsibility has demanding conditions, both control conditions and epistemic conditions, and those agents most likely to require scarce medical resources are also least likely to satisfy these conditions (Friesen 2018; Wikler 2002). Many serious health problems arise from the consumption of addictive substances (principally alcohol and tobacco), and addiction obviously decreases control. Lower socio-economic status (SES) individuals are more likely to suffer health problems requiring care, and such individuals are also less able to access lifestyle-related information, and therefore may satisfy the epistemic condition to a lesser degree than others. As we will show, however, most agents who cause their own ill-health satisfy the conditions on moral responsibility well enough to count as responsible on standard tests.

There are nevertheless reasons to worry about the ‘responsibilization’ (Brown, Maslen, & Savulescu 2019) of health beyond the obstacles to responsible agency that those most likely to need scarce resources face. A number of theorists worry that the emphasis on the responsibility of individual agents for their behavior and its consequences has the deliberate or inadvertent effect of taking pressure off actors who are more powerful and who play a bigger role in producing the very states of affairs for which agents might be blamed. Theorists in this camp argue that those who are most responsible – for crime (Waller 2015), for tobacco use (Mejia et al. 2014), for obesity (Brownell et al. 2010; Kersh & Morone 2002), and for climate change (Supran & Oreskes 2021) – deliberately promote a rhetoric of individual responsibility in order to escape blame themselves, and to continue to engage in practices which are harmful to ordinary people. These theorists argue that we ought to abandon talk of individual responsibility in order to sheet home the blame to those with the most power to change things and the most responsibility for the status quo.

Responsibility is not zero-sum. It is perfectly possible for two or more agents to be fully responsible for one and the same state of affairs without their responsibility being diminished. Compare two thieves who each break into a shop. They seem equally to blame, even if one succeeded only because a disgruntled employee at one shop had disabled the alarm in the hope someone would try to break in (whereas the second shop had no alarm). The thief and the employee are both responsible for the theft, but in this case, responsibility shared is not responsibility reduced. There is no obstacle, in principle, to social agents (governments and corporations) bearing a high degree of responsibility for agents’ ill-health, even while those agents themselves bear (some or a great deal of) responsibility for the same outcomes.

Nevertheless, the apparently warranted appeal to the responsibility of individual agents – warranted because these agents satisfy standard, and justifiable, tests for responsibility – has in actuality been sufficient to block many calls for corporations to minimize the harmful impact of their products and for governments to take steps to improve the health of their citizens. In this paper, we argue that the relative neglect of corporate agency is an expectable outcome of how the responsibility of individual and extra-individual contributors to harms is typically conceived. When we see these sources of harms as competing or interacting causal contributors, the decks are stacked: individual agency, the proximate cause of harms, is most salient and is seen as bearing the lion’s share of the responsibility. We argue that a better understanding of individual responsibility, an understanding that situates it in the context in which it is exercised and understands it as scaffolded *by* social agents, may help to move us beyond the current impasse, in which individual responsibility is seen to block the attribution of any significant degree of social responsibility. Scaffolded individuals may rightly be held responsible, but a recognition of how their agency is scaffolded, and who scaffolds it, will lead to a fairer allocation of responsibilities. In particular, it opens the way to holding corporate agents *prospectively* responsible. That is, it allows us better to recognize the obligations that they have to reduce or mitigate the harms that predictably arise from how they scaffold agency, and the correlative duty of having to bear some of the burdens that such harms entail.

## Responsibility for Health: the Landscape of Debates

Roughly, moral responsibility is causal responsibility that satisfies two additional criteria: a control condition and an epistemic condition (Mele 2010). An agent is morally responsible for something when they exercised sufficient control over bringing it about (it was sufficiently sensitive to actions within their power) and they knew that, how, and why they should exercise that control. The agent in the mouthwash case fails to satisfy the epistemic condition, on the assumption that he had good reason to trust his dentist’s advice. There seems no reason why agents cannot satisfy these conditions with regard to their ill-health. While some people face special barriers to the exercise of control (and some of the causes of ill-health are addictive, which entails reduced control on their users’ part), many of us indulge in unhealthy lifestyles despite satisfying standard tests for the exercise of control and despite knowing that we thereby risk future morbidity and early death.

Individual responsibility cannot alone explain the patterns of morbidity and early mortality observed, however. It is a striking fact that, although many of the agents who cause their own ill-health do satisfy standard (and, we will argue, appropriate) tests for the exercise of control, ill-health has a social gradient. Socio-economic status predicts health outcomes quite well all by itself; if you add in other factors beyond agents’ control (such as the quality of their local environment), its predictive power increases further (Marmot 2018; Marmot et al. 2008). Yet the explanatory mechanism does not *bypass* agents’ control: rather, these factors predict ill-health, in very important part, by predicting *how* agents will exercise control (e.g. Darmon & Drewnowski 2008; Pechey & Monsivais 2016). The different contexts in which agents find themselves, that is, the circumstances in which they choose, predict (in aggregate) *how* they will choose. Individuals do themselves exercise some degree of control over this context, principally by choosing which environments to enter. But they typically have much less control over these facts than do corporate agents. That suggests that these corporate agents bear ultimate responsibility for how individuals act, and makes it tempting to absolve individuals of responsibility. As we will see in the next subsection, however, the case for holding individuals responsible for their own ill-health is strong.

### (a) Individual responsibility

Agents who cause their own ill-heath typically satisfy standard tests for moral responsibility. Agents exercise control over their actions or over outcomes that they can cause by acting (or refraining from acting) when they are *reasons-responsive* (Fischer & Ravizza 2000; McKenna 2013). Tests for reasons-responsiveness and thereby for control have become quite intricate, but we need not enter into their details in this paper. The basic idea is clear enough: an agent exercises control if she would act differently were she given sufficient reason to do so. Control requires reasons *receptivity* – a capacity to grasp reasons as reasons – and reasons *reactivity* – a capacity to respond to them. A typical thief is responsible, at least in part, because she is reasons-responsive. She is almost certainly receptive and reactive to a range of reasons, including moral reasons. Were she confident that she would be caught, she would probably refrain; she is sensitive to prudential reasons. In most cases she would probably refrain were the moral stakes high enough (for example, if every child in an orphanage would die as a result of her actions). If she is not responsive to reasons, including moral reasons, she is not responsible (Fischer 2011). The kleptomaniac who steals *no matter what* acts compulsively and is excused, other things being equal.

In real-life cases, of course, control comes in degrees. A test turning on reasons-responsiveness is able to capture degrees of responsibility. In actuality, there are almost certainly no kleptomaniacs who steal *no matter what*. Even the kleptomaniac is reasons receptive and reactive to some degree. But we can assess whether they have *enough* control to count as responsible (for a particular action in a particular context) by asking about how broad a range of reasons a particular person is responsive to, and how strong these reasons need to be to lead them to act differently. If the kleptomaniac is unable to modulate her behavior in response to reasons that she takes to be decisive (say, public humiliation), she probably does not exercise enough control to count as responsible. If she only responds to extraordinary reasons (like the agent in Mele’s (2000) example of an agoraphobic who would leave the house but only if it were on fire), then she is not responsible for failing to respond to ordinary moral reasons. Some degree of control is not enough for moral responsibility: we need to assess whether an agent has *enough* control to count as morally responsible.

Surprisingly, perhaps, this kind of well-validated test for control actually seems to indicate that not only does the average person with a poor diet exercise sufficient control to count as responsible, so does the typical addict. We tend to think of addiction as destroying control (this is sometimes even built into definitions of addiction (consider Alan Leshner’s (1997) definition of addiction as characterized in “essence” by “compulsive drug seeking and use”). But both experimental and epidemiological evidence indicates that addicts retain a great deal of control – enough, it seems to count as responsible.

Addicts are reasons receptive: except in those relatively rare cases in which addiction causes major cognitive impairment, the agent is perfectly able to understand the negative consequences of future use. More surprisingly, they are reasons reactive; crucially, they are reactive to a range of reasons of a perfectly ordinary sort and strength. For instance, the consumption of drugs is price sensitive, even in the lab and even subsequent to a priming ‘taste’ (Fingarette 1989). Addicts will refuse to work for their drug if they regard the amount of work required as unfair (Elster 2009). Cocaine addicts will refrain from consumption for an extended period of time in exchange for low value vouchers, for things like cinema tickets (Higgins et al. 1994; Lussier et al. 2006). Addicts may deliberately refrain from consumption for a shorter or longer period of time in order to lower their tolerance for a drug and thereby make it cheaper. Most addicts seem to ‘mature out’ of addiction on their own, and they seem to do so in response to ordinary incentives (Heyman 2009). Landing a new job, entering a new relationship or having a baby all provide incentives that prove powerful for many addicts (it should be noted, however, that there may be a relatively small subset of addicts who do not exercise sufficient control over their behavior to satisfy this test).

The counterfactual test for control gives us a satisfying metric that seems to get most cases right. Applying it to agents who are causally responsible for their own need for scarce health resources seems to indicate that they usually possess sufficient control to be morally responsible for their need. While there are surely exceptions, and while we ought to temper justice with mercy in recognizing that some people find it harder to exercise control than others, there seems no particular obstacle to holding people responsible for their own ill-health. In the next section, however, we point to *social* responsibility for ill-health. Should social responsibility crowd out individual responsibility?

### (b) Social responsibility

While the counterfactual test for responsibility clearly indicates that the great majority of us exercise an enormous amount of agency over the course of our lives and our future health, we do not exercise agency in conditions of our own choosing. It is a striking fact that while the details of how we will exercise our agency are highly unpredictable (what will this person have for lunch today?), the overall picture is often quite predictable. There is a social gradient in health outcomes, with individual agency mediating between social conditions and outcomes (Braveman & Gottlieb 2014; Booysen, Botha, & Wouters 2021). While the determining strength of the conditions in which we exercise our agency should not be exaggerated, they are nevertheless powerful – powerful enough for the World Health Organization to describe socio-economic factors and the conditions associated with them as the “fundamental drivers” of health outcomes (WHO 2008). How individuals choose to exercise their agency is, in broad outlines, powerfully shaped by the conditions in which they find themselves, and these are in turn shaped by government and large corporations, and other social actors. That suggests that there is a role for social responsibility in assessing health outcomes and allocating scarce resources.

We can most easily see this by simply applying our test for moral responsibility to the agent or agents that determines the conditions in which people make their choices. The biggest obstacle to such an application is identifying the appropriate agent to whom the test should apply. For a collective agent to satisfy such a test, it must (a) be capable of having beliefs and therefore of satisfying the epistemic condition, and (b) be capable of modulating action in response to reasons. There is every reason to think that it is possible to identify collective agents that satisfy these conditions and to which the tests for control can be applied.

There are deep issues with regard to the nature of the responsibility that can be attributed to collectives (Isaacs 2014; Giubilini & Levy 2018). When a corporation, an army platoon, a club or a sports team (to mention just a random selection of such collectives) brings about a state of affairs and satisfies the epistemic and control conditions on responsibility, is the collective *itself* responsible or is responsibility reducible to the responsibility of key decision makers within the collective? If the collective is itself responsible, how can it effectively be *held* responsible? Does it make sense to inflict a punishment, say, on the collective, or does such a burden necessarily fall only on particular individuals? For many years, the dominant view in philosophy was that collective responsibility was fully reducible to the responsibility of individuals within collectives, in particular those individuals with decision-making power. More recently, however, the pendulum has swung the other way: the currently dominant view is that collectives can satisfy the control and epistemic conditions on responsibility, and in ways that are not reducible to individuals satisfying these conditions (List & Pettit 2011). We endorse this view, because we maintain that collectives have causal powers that individuals within them lack, and that exercising these causal powers requires knowledge that exceeds the knowledge of such individuals.

This is most obvious with regard to control. Collectives can often bring about states of affairs that no individual in the collective is able to bring about. The ceo of a corporation may have little idea how their gizmos are produced and no capacity to distribute them. Indeed, it is typically the case that *no one* in the corporation knows how everything works or could do all, or even more than a very small number, of the jobs that together enable the corporation *to get its product to consumers* (or *to bankrupt its competitors* or *bust the union*). The same holds true with regard to the epistemic condition. It is typically the case that knowledge is distributed across a scientific laboratory, for example, with no individual having the capacity to check the work of many of the others (Hardwig 1985; Winsberg, Huebner, & Kukla 2014). The lab’s findings are appropriately attributed to the entire group. The same is true of the information collating and transforming apparatus of a corporation, from the research and development team to the market research arm.

The success of these collectives in intentionally acting on the world is all by itself good evidence that they satisfy the control and epistemic conditions. States of the world are sensitive to their behaviors, and they have processes for incorporating and responding to information. They are reasons receptive (they might have dedicated departments devoted to gathering information about the state of the market) and reactive (were they to detect falling demand for their product they might reduce its price or increase advertising, for example). Exercising control entails satisfaction of the epistemic condition, because agents can control states of affairs only if they know how these states of affairs are sensitive to their actions (Mele 2010; N. Levy 2011).

Plausibly, agents can be held *morally* responsible for their actions only if they also satisfy the epistemic and control conditions with regard to the harms (and benefits) of their actions (Fischer & Ravizza 2000). We suggest that corporations and governments typically (though not always) satisfy this condition too. The very fact that a corporation fights against sugar taxes or other regulations of their behavior entails their awareness of the relationship between their products and harms, since proponents of such regulations are known to justify them by reference to these harms. While they might not have been aware of the health effects of tobacco, sugar, or fossil fuels when they first began to market them, the time when they could claim ignorance is long past. Indeed, in all three cases there is plentiful evidence that corporations have engaged in deliberately deceptive propaganda to minimize such harms (for plentiful examples of knowing deception by tobacco and fossil fuels companies, see Oreskes and Conway (2011); Milman (2023) cites more recent evidence of deliberate deception).

Governments, too, knowingly exercise a significant degree of control over the health of their citizens and residents. Governments (or key individuals who comprise them) know that the health of citizens is responsive to the conditions that they find themselves in and that these conditions are in turn sensitive to the actions of governments. Indeed, most governments explicitly act on this knowledge to improve the health of their citizens. Very many governments tax tobacco products and alcohol at a higher rate than many other consumer items, and justify these measures on public health grounds (Wilson & Thomson 2005; Ho et al. 2018). These taxes are supposed to disincentivize consumption and are often accompanied by public health campaigns. Similarly, some countries have introduced ‘sugar taxes’ which are designed to make unhealthy foods relatively more expensive and thereby encourage better diets (Fernandez & Raine 2019; Véliz et al. 2019). Again, these may be accompanied by public information campaigns (e.g. the UK ‘5 a day’ campaign, aimed at increasing the consumption of fruit and vegetables). Some nations have introduced networks of cycle paths to encourage more active lifestyles and to reduce pollution (and thereby its ill-effects on health).

While it is very difficult to disentangle the causal contribution these campaigns have made from other influences, it is overwhelmingly likely that they have had some effect. Available evidence strongly supports the effectiveness of taxation measures and of public health campaigns (D. T. Levy et al. 2016; Hoffman & Tan 2015). Thereby, governments have shown that they exercise a significant degree of control over the health-related behavior of citizens. They know that they exercise this control, how they exercise it, and understand the stakes of doing so. They thereby seem to satisfy the conditions on moral responsibility for the health of their citizenry.

Importantly, large segments of their population continue to engage in unhealthy behaviors and there is every reason to believe that governments could do more to prevent this. The measures that they have put in place are often low-hanging fruit. It is easy and tempting to implement ‘sin taxes’ like taxes on sugar and tobacco – easy because those who are most subject to them often lack much political power and may additionally be stigmatized – and tempting because of course such taxes provide a source of revenue to governments. Governments could do much more, though, to improve health if they were willing to make hard political choices to limit the power of corporations, to restrict certain sorts of advertising, to expend resources to ensure that the residents of low-income neighborhoods are offered more opportunities for outdoor recreation and fewer opportunities for unhealthy behaviors that they would prefer to avoid if given other attractive, healthier options.

We suggest that the current policy debate, and to a large degree the debates within philosophy and applied ethics, are unhelpfully polarized between these two conceptions of responsibility, and – moreover – that the salience and proximity of individual agency ensures that it dominates discussion and thought. In public debate, corporations and their political allies argue that governments and business are not required to take steps to limit the harms that arise, directly or indirectly, from their products and activities *because individuals are responsible*. This is a powerful move, and not merely rhetorically: those who advocate for more action on the part of institutional actors cannot plausibly deny that individuals *are*, typically, responsible. Even when we acknowledge that patient responsibility does not preclude the responsibility of supra-individual actors, the fact that individuals typically fully satisfy plausible tests for responsibility for their behavior is widely felt to place the onus on them. That is, they are widely held to be sufficiently responsible for their behavior to bear the costs of remedies, and to have the responsibility to avoid these costs in the first place, even if other actors are also partly responsible (Everett et al. 2021).

Inside applied ethics, philosophy, and medicine itself, attempts to avoid the resulting impasse have had little success. One approach has been to attempt to avoid the issue, at least when it comes to the bearing of costs and to priority for organ transplants, by using prognosis rather than responsibility as a deciding factor. This has the happy result (by the lights of many) of deprioritizing those who might be seen as responsible for their need on grounds *other than* responsibility: that is, on the grounds that they can be expected to return to problematic behaviors and therefore have a worse prognosis than those whose need arose blamelessly. In practice, however, the use of prognosis as a criterion all too often smuggles in responsibility: perceptions of prognosis are influenced by the attribution of responsibility, among both the general public (Ubel et al. 2001) and medical professionals (Everett et al. 2021). Moreover, the focus on prognosis does not aid us when it comes to assessing whether institutional actors should bear some responsibility for harms and be required to change their behaviors. At worst it distracts from the issue. At best, it is silent on it.

The last problem also arises with a final suggestion: that we apply a strict liability model to bearing the costs of harms. Such a model is most plausibly applied to patients, who are the most salient and most proximate cause of the burdens that they bear. To that extent, the strict liability model converges with a responsibility model. We might argue that strict liability applies to *both* individual agents and to corporate agents: They are both ‘but for’ causes of the harms that individuals suffer. That is plausible, but leaves us no further along when it comes to the question of who should bear what costs. Corporate agents might argue that since their actions do not entail harms unless individuals consume their products excessively or recklessly, they should not be forced to pay the costs that are associated with them. More seriously, once again perceptions of individual moral responsibility are likely to influence how we assess the relative contribution of each set of actors.

So long as we continue to think of corporate agents and individuals as competing and interacting sources of responsibility, there seems to be no principled way of settling which set of agents should bear what costs. Moreover, because individual agency is the proximate and most salient cause of the harms, we will tend to err on the side of making individuals bear the resulting costs. We will argue that individual and social responsibility are not the only options available, however. There is a third option, an option neither entirely individual nor entirely collective. We turn to that kind of responsibility next.

### (c) Scaffolded responsibility

An alternative to seeing responsibility as divided between collectives and individuals is to see collectives (and, indeed, other agents) as supporting or undermining the responsibility of individuals (Holroyd 2018). We can say that agency is *scaffolded* when it is partially dependent on supports of various kinds external to the agent. These supports – scaffolds – may be temporary or permanent, essential or merely helpful. Training wheels on a bicycle are temporary scaffolds of agency: all going well the learner will dispense with them as she gains in skill. The wheels may transition from essential to helpful: at first the learner may be unable to ride without them, but as she gains experience, she may rely on them more for confidence than for remaining upright. Some scaffolds are not designed to be dispensed with. A cane may be integrated into a blind person’s agency, and she may come to be increasingly skillful at navigating the world thereby. The cane is not intended to bring her to a state in which she can dispense with it.

Scaffolds can support knowledge (and thereby the epistemic condition on responsibility) just as much as they can support abilities. Consider roads. They can be seen as scaffolding our navigational capacities. An agent does not need to map out the way from one town to the next: she can just follow the road. She can rely on road signs not only to convey explicit information but also to alert her to features of the road worth attending to. In developed countries, at least, agents do not need to keep a lookout for dips or unobvious obstacles, because they can rely on the authorities both to repair problems and also to signal the presence of those that cannot be avoided. We can navigate the world and pursue our goals effectively in very important part because the world comes prestructured to make it easier to identify effective means to our ends (at least if our ends are widely shared) and to carry them out (Ramstead, Veissière, & Kirmayer 2016).

But if agency can be supported through scaffolding, it can also be undermined through scaffolding. The world is deliberately prestructured so that certain goals are easier to pursue and certain information more easily attained. But the world is also prestructured so that certain goals are harder to pursue and certain facts harder to know. To some extent, this is the simple corollary of scaffolding agency: scaffolding certain ways of behaving entails making some other ways more difficult (roads scaffold navigation but they also make it more difficult to travel across, rather along, the road – as the fate of roadkill illustrates). In other cases, undermining agency may occur through sheer neglect, rather than as the obverse of scaffolding other ways of behaving, and in yet other cases scaffolding is malign or at least reflects ends that are not those of the agents whose behavior is scaffolded. It is plausible that some of the behaviors of individuals, especially those in lower SES groups, are made more likely by the ways in which their agency is undermined and unhealthy ways of behaving are scaffolded.

Consider, for example, the distribution and density of fast-food restaurants. Such restaurants are often concentrated on the high streets of lower-income areas (Thornton, Lamb, & Ball 2016; Hilmers, Hilmers, & Dave 2012; Public Health England 2017). They offer cheap and of course quick meals which are tempting for those who are time and income poor. Their clustering makes it harder to avoid them and easier to choose them. Their bright signs and lights make them more salient to agents. In contrast, the supermarket may be less accessible and of course fresh food bought there is less available, inasmuch as it takes time and processing to transform it into a meal. For these reasons, it seems reasonable to say that choosing the unhealthy option is scaffolded and the choice of better options made more difficult by the way in which the world is structured.

Similarly, American cities which lack sidewalks scaffold driving, and undermine the capacity to walk by making driving relatively more accessible. Road laws may undermine the choice to cycle by making it relatively more dangerous or by exposing the cyclist to pollution. Advertising (for alcohol, for example) makes certain choices more salient and thereby scaffolds knowledge about their availability. Rather than seeing individual agency and collective agency as potentially competing sources that account for the outcome – the ill-health of certain individuals – we might instead see them as interacting, with collective agency scaffolding or undermining how individual agency is exercised. Seeing individual agency as scaffolded in this manner helps to explain the observed pattern of differential outcomes in morbidity and mortality predicted by SES. Healthy agency by some people and the unhealthy choices of others are scaffolded by the ways in which the world is prestructured, with that prestructuring produced by the choices of government, corporations and other collective entities.

While few people will deny that individual choice is scaffolded by collective entities, some will worry that we are introducing new terminology for an existing concept. Thaler and Sunstein (2008) have influentially argued that policymakers should *nudge* agents into making better choices. To nudge an agent is to modify the ‘choice architecture’ – that is, the context in which choices are made – to encourage better choices. Nudges are inspired by work in the behavioral sciences, allegedly showing that small changes to the choice architecture, such as changing defaults or making certain options more salient, may have significant effects on behavior (note, however, that there is an ongoing dispute over whether nudges have effects which are significantly different from zero (Szaszi et al. 2022; Mertens et al. 2022)).

While we think that nudges work, when and if they work, by scaffolding agency, we deny that we are simply changing terminology. First, the scaffolding of agency refers to ways in which agency is supported in ways that extend beyond nudging. Scaffolds need not make options salient to the agent; they can support us in making our choices without always recommending options. Two-way scaffolds, that allow agents more easily to pursue the healthy *and* the unhealthy option, for example, are perfectly feasible. A social media site might make partisan news that is congenial to two competing sides equally salient, or it might nudge choice in one direction. Second and more importantly, we think that the concept of nudge recapitulates, rather than avoids, the problems that arise from thinking of individual and collective agents as competing and interacting sources of responsibility.

This is most clearly brought out by the ongoing debate over whether nudges are paternalistic. On the one hand, those who object to nudges on these grounds see them as interfering with, rather than supporting, agency (Bovens 2008; Wilkinson 2013). On the other hand, Thaler and Sunstein’s response to this charge itself highlights individual agency: they argue that the nudge program exemplifies ‘libertarian paternalism’ since it leaves all options available to the agent. This debate might illuminatingly be seen as turning on how much of individual agency nudges preserve, and highlights the degree to which individual and extra-individual agency are seen as competing forces when behavior is nudged. Seeing agency as scaffolded, rather than nudged, brings out how agency is supported by extra-individual forces (for better or for worse) rather than competing with it. We argue that thinking of agency in this sort of way avoids the impasse that we currently face.

## Whose Responsibility?

Too often, debates over public health initiatives pit those who advocate structural or policy changes on the grounds that responsibility is primarily or solely the province of corporate agents against those who instead emphasize individual responsibility. The former point to the social determinants of health, including the correlation between the opportunities and temptations that a particular environment makes available, and argue that effective intervention must change these facts external to the agent. The latter insist, reasonably, that lower SES individuals (and others who may predictably choose badly) are not coerced to act as they do, and that better choices remain available to them. Proponents of individual responsibility can point to standard tests for moral responsibility to make their point. While there are surely agents who cause their own ill-health who fail such tests, the great majority do not. Most possess enough knowledge and are sufficiently responsive to reasons to pass such tests, often easily. How they exercise their agency is predictable, but it remains *their* agency that is exercised. The fact that *some* individuals exercise their agency well in the same contexts in which many choose badly is strong evidence that reasons-responsive agency is at work.

Responsibility is not zero-sum. But corporate agents have been very successful in forestalling calls for them to take action, and policies that would rein in their capacity to shape individual agency, by appealing to individual responsibility. Recognition that individuals who cause their own ill-health thereby exercise their agency is sufficient for the public to perceive that it is the same individuals who must bear the majority of any responsibility (Lund, Sandøe, & Lassen 2011; Hardus et al. 2003; Wolfson et al. 2015). The recognition of individual responsibility effectively blocks the attribution of a significant level of blame to the corporate agents who structure individual agency.

Appeals to personal responsibility are often heard when critics of big business (in particular) call for more regulation. Such appeals often serve a political end: they aim to forestall effective regulation (Friesen 2018). They would not succeed did they not exercise some intuitive grip on our imagination (Wikler 2002; Steinbrook 2006). We suggest that the appeal to individual responsibility would not block attribution of a significant level of responsibility to corporate agents (and other social agents) when it is an appeal to scaffolded agency. Scaffolded agency is individual agency – it is no one but the person herself who exercises it – but it is individual agency structured by and leaning on the affordances and the features of the environment that others have provided. Rather than seeing individual agency as competing with corporate agency for control of choice, we should see individual agency as scaffolded by corporate agency. We think that conceiving of agency in this manner promises to break out of the impasse that characterizes current debates over the responsibility of extra-individual agents, and thereby to promote better outcomes for individuals and society.

Seeing individual and corporate agency as competing for control of choice stacks the decks: since individuals are the proximate and most salient causes of their own behavior, their contribution to the harms will tend to block serious consideration of the role that corporate agents play. When individual choice is necessary for harms to arise, and the individuals in question satisfy plausible tests for full responsibility for their choices, their agency will be seen as the most important contributor to any resulting harms. If individual and corporate agency are seen as competing for control of behavior, individual agency wins every time. Ironically, this is a win that may come at a cost to the individuals who exercise this agency: ‘winning’ this competition ensures that the onus is placed on them to change their behavior and the burdens of addressing resulting harms are theirs to bear. In contrast, seeing individual agency as scaffolded respects the contribution that agents make, thereby respecting their autonomy, but emphasizes how they choose in circumstances that are not of their own making and that all too often do not support them in choosing well.

Importantly, seeing agency as scaffolded highlights the role of the scaffolding, and thereby opens the way to attending how it might be scaffolded better. While we do not intend to offer policy prescriptions, we suggest that attention to agency as scaffolded might be more productive when it comes to holding corporate agents to account. We should no longer ask *who is responsible for these choices*? but *are corporate agents scaffolding agency well*? The answer to the first question need not imply an answer to the second: perhaps individuals are fully responsible for their own ill health *and* corporations have a responsibility to scaffold agency such that bad outcomes are avoided far more often than they currently are. Corporations cannot easily shirk this responsibility by highlighting individual agency and responsibility: in doing so, they cannot but draw our attention to the scaffold, as well as to the agent.

We therefore suggest that conceptualizing agency as scaffolded will enable us to break out of the current impasse, where those agents who shape the conditions in which individual agency is exercised can deflect calls for more effective regulation of markets, for targeted taxes, for the provision of green spaces and sidewalks, and so on, by highlighting that individual agency. These features scaffold individual agency, and making individual *scaffolded* agency salient cannot but bring these features to our notice. We therefore suggest that an appeal to scaffolded agency may lead to a fairer allocation of the burdens associated with addressing ill-health. We will not attempt, here, to apportion burdens (such apportioning must in any case be sensitive to many properties beyond responsibility, such as the ability to pay). We limit ourselves to pointing out that since scaffolded agency is genuine agency – indeed, *all* agency, including the most admirable, is scaffolded – there is no reason in principle to think that the fact that their agency is scaffolded prevents us holding individuals responsible, to some degree. But the fact that it *is* scaffolded opens the way for holding corporate and other institutional agents responsible as well: in practice; that is, it opens the way to requiring them to take steps to mitigate or avoid the harms that predictably arise from how they scaffold agency now.

## Conclusion

The academic literature, public debate and government policy often appeal to personal responsibility for health. There is persuasive evidence that these appeals lead people to allocate responsibility to individuals *rather than* corporate agents. We believe that this is unfortunate, insofar as it prevents effective measures addressing ill-health and health inequalities from being taken. We suggest that the broader recognition that individual agency is scaffolded, and that corporate agents play a central role in constructing these scaffolds, might lead to better outcomes. Scaffolded agency is genuine agency and in exercising it agents satisfy standard tests for responsibility. We may rightly hold them responsible for their choices and the outcomes of their choices. But scaffolded agency is structured by forces beyond individual control and to that extent reflects the choices of other agents. Scaffolded agency reflects individual choice and corporate structuring of that choice, and leads to a better allocation of responsibility for such choices. To that extent, its recognition may open the way for new and better policies.
